# Production and Multiplication of Native Compost Fungal Activator by Using Different Substrates and Its Influence on Growth and Development of *Capsicum chinensis* Jacq. “Bhut Jolokia”

**DOI:** 10.1155/2015/481363

**Published:** 2015-01-06

**Authors:** Vipin Parkash, Ankur Jyoti Saikia

**Affiliations:** Mycology and Soil Microbiology Research Laboratory, Rain Forest Research Institute (ICFRE), An Autonomous Council of Ministry of Environment & Forests, Government of India, Deovan, Jorhat, Assam 785001, India

## Abstract

*In vitro* experiment was carried out to see the effect of saw dusts of *Pinus kesiya, Shorea robusta*, and *Callicarpa arborea* on *Trichoderma harzianum*, isolate TH-13 mass production, along with its biotization effect on *Capsicum chinensis* Jacq. “Bhut Jolokia.” Early mycelium initiation (2 days) occurred in *S. robusta* followed by *P. kesiya* and *C. arborea* (3.5 days). The sporulation was observed earlier in *S. robusta* (100% after 6 days) than *P. kesiya* (33.4% after 8 days) and *C. arborea* (16.7% after 9 days) but no sporulation was observed in control. The complete sporulation was also earlier in *S. robusta* (100% after 10 days) than *P. kesiya* (33.4% after 15 days) and *C. arborea* (16.4% after 18 days). Higher conidial yield (86 × 10^6^) was also in *S. robusta* than *P. kesiya *(70 × 10^6^) and *C. arborea *(45 × 10^6^), respectively. The increase in height (60–70 cm), number of leaves (600–650), and yield of chili (120–150 fruits) were also more in inoculated *C. chinensis* seedlings than control. It is concluded that *S. robusta* saw dust is the best substrate for mass production of compost fungal activator and can be used in nursery practices for quality stock production of various crops/plantations.

## 1. Introduction

Compost is a mixture of decayed organic material decomposed by microorganisms in a warm, moist, and aerobic environment that release nutrients into readily available forms for plant use. Among these microorganisms, fungi like* Trichoderma* spp. Pers. are important microbes that help in decomposition of organic material and are known as “compost fungal activator” (CFA).* Trichoderma* spp. produce hyphae which are branched, creeping, forming a tuft or cushion on natural substrate, septate; conidiophores are erect, indefinite, arising from short branched side branches, bearing phialides laterally and terminally; phialides are surrounded by heads, rarely by short chains of slime spores; conidia are hyaline or bright coloured, one celled and chlamydospores are terminal or intercalary [1].

Mass production of* Trichoderma* spp. has become a focus of research in the search for alternatives to polluting pesticides and chemical fertilizers for control of plant diseases and growth promoter of plants. The majority of commercially available biocontrol fungi available in the world are of* T. harzianum* Rifai origin only and their efficiency is well established. Techniques for large-scale biomass generation of this fungus are still in infancy. Consequently any media used for mass production of* Trichoderma* spp. must be economical and able to support production of large quantities of biomass and available propagules. A variety of media have been used by various researchers for production of* Trichoderma* spp. in stationary flasks [2], shakers [3, 4], and liquid fermenters [5]. Molasses-brewer's yeast medium is being used widely for commercial production of* Trichoderma* spp. by fermentation process [6]. In India, many commercial* Trichoderma* producers are using molasses-yeast medium for mass production. Brewer's yeast which serves as nitrogen source is expensive and there is a need to identify inexpensive and more commonly available alternative to yeast fungi.

Jin et al. [7] developed a medium from modified Richard's medium + V-8 Juice (RM8) to produce high level of desiccation tolerant conidia of* T. harzianum* strain 1295-22. The addition of 9 percent (v/v) glycerol to RM8 medium improved both biomass production and desiccation tolerance of conidia. Several commercial formulations of species of* Trichoderma* have been made [8]. For commercial production, it is essential that the product should possess certain qualities. The dose requirement should be minimum, must have large shelf life, is easy to apply, is free from contamination, and has economic feasibility with positive monetary return.

*Trichoderma* spp. are mostly lignocellulose decomposer. Lignin protects cellulose, hemicelluloses, and carbohydrate in lignocellulosic materials. Lignocellulolytic microorganisms are the key agents in depolymerizing the lignin barrier in organic materials. Therefore, the selection of effective lignocellulolytic microbe/s is a crucial step leading to the success in accelerating composting of lignocellulosic materials.* Trichoderma* spp. are widely known as a lignocellulose decomposer as already explained above because they are filamentous and have the ability to produce prolific spores which can invade substrates quickly [9]. Various studies have shown that composting of lignocellulosic materials preinoculated with potential* Trichoderma* spp. can reduce the time of biodegradation [10].* T. harzianum* was used as inoculant to enhance composting of rice straw and weeds [11]. However, there is no or a little work that has been done on composting of different saw dusts using* Trichoderma* spp. (CFA) isolated from the related ecological habitats. Hence, a study is carried out to analyze the different substrates (saw dusts) suitability for mass production of CFA along with its effect on growth and development of* Capsicum chinensis* Jacq. “Bhut Jolokia”.* C. chinensis* Jacq. “Bhut Jolokia” is being cultivated and consumed in different states of Northeast India including Assam, Nagaland, Manipur, and Mizoram since antiquity. It is either used as a spice in food or eaten raw along with the staple food. On account of its refreshing aroma, palatability, and medicinal properties, the populace has been using it for pickle preparation, flavouring curries, apart from its usage for home remedies of ailments like gastritis, arthritis, and chronic indigestion problems. It is also used as a remedy to summer heat, presumably by inducing perspiration [12]. To analyze the influence of CFA on growth and development, “Bhut Jolokia” was selected as it is cultivated by the local people in their homestead gardens as a spice and CFA technology is extended to Demo village of the Institute at Meleng Grant, Jorhat, Assam, as cited below in nursery trials in Material and Methods.

## 2. Material and Methods

### 2.1. Collection of Soil Samples and Isolation of Compost Fungal Activator

**Figure 1:**
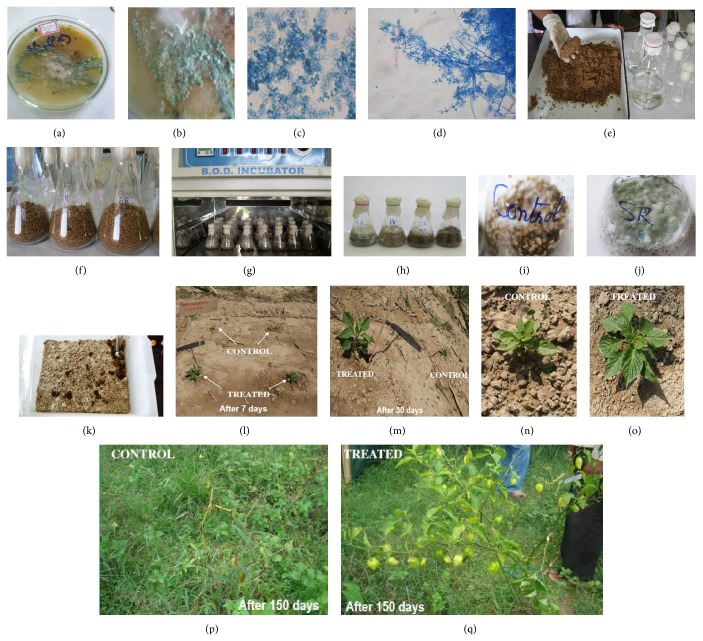
(a) Culture of* Trichoderma harzianum* (isolate TH-13); (b) mycelial mat of* T. harzianum*; (c) spores of* T. harzianum*; (d) hyphae/mycelia of* T. harzianum*; ((e), (f)) preparation of substrates and filling of flasks; (g) incubation of flasks at 27°C in BOD incubator for 6–8 days; ((h), (i), (j)) mass production trends of* T. harzianum* on different substrates; (k) mass production of* T. harzianum* in a tray; (l) variation in treatments on* Capsicum chinense* “*Bhut Jolokia*” after 7 DAI; ((m), (n), (o)) variation in treatments on* C. chinense* “*Bhut Jolokia*” after 30 DAI; (p) a control plant after 150 DAI; (q) a CFA treated plant after 150 DAI.

The soil samples were collected from Nongkhyllem Reserve Forest, Nongpoh, Meghalaya, India. Fungal species* Trichoderma harzianum* was isolated from the soil samples by using serial soil dilution method [13] on potato dextrose agar (PDA) medium. The inoculated plates were incubated at 30°C for 4 days. The pure fungal colonies were picked up and purified by streaking on agar slants and incubated at 30°C for 7-8 days. Green conidia forming fungal bodies were selected and microscopic observation was done and the fungus was identified to be* Trichoderma harzianum* (isolate/accession number TH-13). The preserved fungal isolate/culture maintained on PDA slants is retained with Mycology and Soil Microbiology Laboratory, Rain Forest Research Institute, Jorhat, Assam, India, for further study (Figures 1(a)–1(d)).

### 2.2. Preparation of Solid Substrate Media

In this experiment, different saw dusts like* Pinus kesiya* Royle ex. Gordon,* Shorea robusta* Gaertn., and* Callicarpa arborea* Roxb. were taken for evaluation. The different saw dusts were shade dried. The dried saw dusts were mixed with wheat bran by adding sterilized water in the ratio (wheat bran : saw-dust : water; 3 : 1 : 4 w/w) as explained above. The moisture level of the mixture was maintained up to 50–60%. The substrate was sterilized through Autoclave (Labotech, BDI-81 make, India) at 120°C and 15 lbsi (Figure 1(e)).

### 2.3. Mass Multiplication of* Trichoderma* Inoculum

The inoculum of* Trichoderma harzianum* was grown on synthetic PDA (Potato Dextrose Agar) medium (SRL, India) for 7-8 days and incubated at 27–30° ± 1°C (Figures 1(f)-1(g)). The inoculum was kept in B.O.D. incubator (Labotech, BDI-55 make, India) for 10–12 days for maximum growth and sporulation. Then the inoculum containing medium was cut into small discs and was put in flasks containing wheat bran and different saw-dust medium in the ratio (3 : 1 : 4 w/w) for mass production of* Trichoderma harzianum*. Approximately 50 g of substrate was taken in 500 mL conical flasks and inoculated with 5 mm mycelial mat incubated at 28°C incubator for 7–10 days earlier. In control set, no saw dust component was added to the substrate. Six replicates of each treatment were taken. The colony forming units were calculated with help of the following formula through serial dilution of one gram of substrate and the results are expressed as cfu g^−1^ mL^−1^ of suspension of each substrate:

(1) $$\begin{array}{c} \text{CFU}/\text{g}/\text{mL}=\frac{\text{Number}\;\;\text{of}\;\;\text{colonies}\;\;\text{per}\;\;\text{mL}\;\;\text{plated}}{\text{Total}\;\;\text{dilution}\;\;\text{factor}}. \end{array}$$

### 2.4. Nursery Experiments/Trials

The substrate supplemented with saw dust of* Shorea robusta* which proved to be efficient in production of more cfu g^−1^ mL^−1^ was selected for inoculation experiment. The target bioagent in the form of substrate inoculum was applied at the time of sowing of seedlings in the nursery of Rain Forest Research Institute's Demo village at Meleng Grant, Jorhat, Assam, located at a distance of 10 km east of Jorhat city on NH-37 (on Jorhat-Tinsukia Highway, Assam, India (26°46′53′′N 94°17′29′′E, 107 m asl)). The annual average precipitation is 500 mm and the annual average temperature is 26°C. The experiment was conducted using field soil supplemented with substrate containing the target bioagent in the field condition. In control rows, no bioagent (i.e., inocula/substrate) was added. The experiment was designed in a single inoculation treatment. Ten replications of each treatment were taken. The following design of the experiment was adopted:

 
*T*_0_ = control (no inoculation),
 
*T*_1_ = treatment/inoculation of* Trichoderma harzianum* (isolate TH-13 supplemented substrate).

Data on plant growth like increase in height (in cm), number of leaves, and total fruit yield (in number) per plant were observed after 150 DAI (days after inoculation) and analyzed to evaluate the efficacy of the preferred bioagent on the growth and development of target seedlings. The data were analyzed statistically for calculating standard error of mean and coefficient of variance (CV) percent by using MS Excel version 2003 and Gupta [14].

## 3. Results and Discussion

### 3.1. Mycelial Growth and Conidial Yield

Different saw dusts (forestry/industry byproduct) were tested as a substrate constituent along with wheat bran for the mass production of* Trichoderma harzianum* (isolate TH-13) micropropagules as shown in Table 1. After 2 days of incubation at 27°C ± 1, the mycelial growth initiation was seen in substrate supplemented with saw dust of* Shorea robusta* while in substrates with* Pinus kesiya* and* Callicarpa arborea*, mycelial growth appeared after 3.5 days.

In control set, mycelia appeared after 5 days. The initiation of sporulation of bioagent was observed earlier in substrate of* S. robusta* (after 6 days) than* P. kesiya* (after 8 days) and* C. arborea* (after 9 days). The completion of sporulation of fungal activator was also observed earlier in substrates of* S. robusta* after 10 days (100%) than* P. kesiya* after 15 days (33.4%) and* C. arborea* after 18 days (16.7%) (Figures 1(h)–1(j)).

No sporulation was observed in control set. The enhanced conidial yield was also higher in* S. robusta* (86 × 10^6^) than* P. kesiya* (70 × 10^6^) and* C. arborea* (45 × 10^6^) substrates in terms of colony forming units (cfu g^−1 ^mL^−1^), respectively. The screening of various substrates for their potential to support* T. harzianum* (as CFA) mass production indicated that, among the tested saw dusts,* S. robusta* saw dust was found suitable in terms of production of high conidial yield and early conversion of substrate into compost. This substrate supplemented with CFA was taken for further inoculation experiment (Figure 1).

Fungi are one of the major agents of decomposition. Estimating fungal biomass in waste is important for understanding quantitatively their parts in the decomposition processes and the nutrient cycling in terrestrial ecosystems.* Trichoderma* and* Gliocladium* spp. are the mostly worked out antagonists, so far the production and delivery system is concerned [15, 16]. So their mass production is essential requisite for bioinoculating the crops.

The substrata used for mass production of inoculum of* Trichoderma* spp. are wheat straw, sorghum grains, lignite and stillage, molasses and brewer's yeast, and wheat bran medium [15–19]. Studies have been carried out to evaluate various agroindustrial wastes including wheat straw, paddy straw, shelled maize cob, paper waste, and sugarcane baggase for mass multiplication of* T. harzianum* through solid state fermentation technology. In order to enhance growth rate and sporulation, various cellulosic residues were also supplemented with chickpea flour as organic nitrogen supplement at 2 and 4 percent. Supplementation of all the substrates with chickpea flour enhanced growth and sporulation. However, 4% chickpea flour supplementation gave maximum response in all the cases. Paddy straw followed by sugarcane baggase and wheat straw, each supplemented with 4% chickpea flour, were found to be promising substrates for mass multiplication of* T. harzianum* [20]. Sterilized oat seeds were found effective for large-scale production of* T. harzianum* [21]. Dhingra and Sinclair [22] also reported that dehulled broken rice grains were found to be an excellent growth and delivery substrate for* T. harzianum* and* T. koningii*.

In this investigation,* Trichoderma harzianum* is cultured only on wheat bran and different saw dusts which are waste materials by saw mills for mass production. It is reported that the sporulation of fungal activator was observed earlier and more in numbers in substrates consisted of saw dust of* Shorea robusta* than* Pinus kesiya* and* Callicarpa arborea*. However, the production of spores of CFA is more in these substrates than control set where no saw dust was added. Noteworthy is the fact that lignin content of test saw dusts which was observed to be directly proportional to the conidial yield of CFA. Maximum sporulation (86 × 10^6^ cfu g^−1 ^mL^−1^) was observed in the media fortified by* S. robusta*, where saw dust contained maximum (536.52 ± 16.6 mg g^−1^ dry weight of wood) lignin content [23], which was followed by* P. kesiya* (70 × 10^6^ cfu g^−1^ mL^−1^) with comparatively less (235.43 ± 2.47 mg g^−1^ dry weight of wood) lignin composition [24]. On the other hand, least conidial yield (45 × 10^6^ cfu g^−1^ mL^−1^) was observed in* C. arborea*, in which lignin content was 25.5% of cortical dry weight [25].

Addition of* T. harzianum* into organic media like neem cake, coir pith, farmyard manure, and decomposed coffee pulp caused an immediate increase in the population up to three days. Soil amendment with organic materials like neem cake, coir compost, farmyard manure, and* Gliricidia* leaves showed better growth and survival of antagonist than soil alone [26]. Previous reports suggested that addition of inorganic forms of nitrogen increases the production of fungal biomass [27]. Serrano-Carreon et al. [28] had been able to prove that* Trichoderma* spp. prefer the ammonium form of nitrogen.

According to Elad et al. [29], biocontrol by* Trichoderma viride* is dependent on the type of inocula or substrate. Different substrate treatments based on combination of substrate like wheat bran, pulse bran, sugarcane baggase, rice straw, wheat straw, cow dung, poultry manure, groundnut shell, and saw dust with tap water are evaluated for mass multiplication of* T. harzianum* and* Gliocladium virens*. Many workers used wheat bran as substrate for mass production of* Gliocladium* spp. and* Trichoderma* spp. [29–33]. Sorghum grains were also used for mass production of* T. harzianum*. These infected sorghum grains served as bait for growth and multiplication of* T. harzianum* in soil [16].

A standard preparation of* T. harzianum* and* T. koningii* by Mehrotra [34] was in wheat bran saw-dust medium which can be very easily grown in autoclavable plastic bags of different sizes. The preparation is ready for commercial use within fifteen days and is stored at room temperature. In the present investigation also, media prepared by combining wheat bran and saw dust/s resulted in maximum yield of biomass, viable propagules, and spores. However, saw dust as a constituent of substrate was used by previous workers, but through this study it is revealed that the saw dust that has more lignin content is preferable for mass production of CFA (*Trichoderma* spp.). In this experiment wheat bran acted as carbohydrate source and saw dust served as medium for moisture for mass production of* Trichoderma* species.

### 3.2. Biotization Effect on Growth Parameters

The influence of* in vitro* mass produced native compost fungal activator (substrate supplemented with CFA) was also observed on growth parameters of* Capsicum chinensis* Jacq. “Bhut Jolokia” in open field experiments, consequent to 150 days after inoculation.

The datum, as depicted in Table 2, obviously explains the significant increase in the growth parameters. The increase in height (60–70 cm), number of leaves (600–650), and total fruit yield (in numbers) (120–150 chili fruits) was also reported more in inoculated plants with bioagent than noninoculated control plants (height, 20–30 cm, leaves, 400–500, and chili fruits, 50–60), respectively (Figures 1(l)–1(q)).

*Trichoderma harzianum* has been reported to promote plant growth [35]. Various strains of* Trichoderma* have been founded to be effective in plant growth characteristics and enhance biomass production [36]. These fungi inhabit plant roots and promote plant growth characteristics by increasing evolution and production of some organic acids in the rhizosphere such as gluconic, citric, and/or fumaric acids by* Trichoderma* which decrease soil pH and lead to increased solubility of the insoluble compound and an availability of micronutrient, as well as an increase in plant nutrient uptake [37, 38]. Improvement of plant nutrient uptake and its transport from root to aerial parts, together with the produced plant stimulators, might result in higher photosynthetic rates [39] required for producing enough energy used to derive the enhanced growth response [38]. The CFA inoculation is observed to increase shoot height, number of leaves, and total fruit yield (in number) per plant in the present study in accordance with the results which were obtained by previous workers on various plants [38, 40].

It would be beneficial to prepare a composted wheat bran and saw dust (that must contain high lignin content) substrate that may serve as a nutritionally rich biodegradable product to be effective in improving the growth and development of chili plants possibly by improving the organic content of soil. The composting of organic wastes with the help of microbial inoculant/s not only helps in recycling of waste but also results in the preparation of economic and environmental friendly organic biofertilizer that could provide benefits to agriculture/horticulture/forestry crops. This technology of bioinoculants should also be extended to other economically and medicinally important plant/s to check their potential to increase the bioactive constituents and metabolites which is a future prospective.

## Figures and Tables

**Table 1:** Effect of substrate (type of saw dust) on* Trichoderma harzianum* mass production.

Substrate (3 : 1) (saw dust : wheat bran)	Mycelium initiation (in days)^*^	Initiation of sporulation (in days)^*^	Completion of sporulation (in days)^*^	Number of replicates completing sporulation	Percentage of sporulation (%)	Colony forming unit (cfu g^−1^ mL^−1^)^*^
A	3 ± 0.00	8 ± 0.00	15 ± 0.00	2/6	33.4	70 × 10^6^ (±0.01)
B	2 ± 0.00	6 ± 0.00	10 ± 0.00	6/6	100	86 × 10^6^ (±0.02)
C	3 ± 0.00	9 ± 0.00	18 ± 0.00	1/6	16.4	45 × 10^6^ (±0.00)
Control	5 ± 0.00	—	No sporulation	—	—	—
CV (%)	—	—	—	—	—	0.01

A: *Pinus kesiya*: wheat bran, B: *Shorea robusta*: wheat bran, and C: *Callicarpa arborea*: wheat bran.

^*^Average of six replicates; CV = coefficient of variance.

**Table 2:** Effect of* Trichoderma harzianum* inoculation on growth and development of *Capsicum chinensis* “Bhut Jolokia” after 150 DAI.

Treatments	Increase in height (in cm)^*^	Number of leaves^*^	Total fruit yield (in number) per plant^*^
Inoculated	65.0 ± 4.08	627 ± 4.96	130 ± 2.49
Control	25.3 ± 4.10	426 ± 3.74	55 ± 4.08
CV (%)	11.24	0.83	4.66

^*^Average of ten replicates; CV = coefficient of variance.
